# Perceived Surface Slant Is Systematically Biased in the Actively-Generated Optic Flow

**DOI:** 10.1371/journal.pone.0033911

**Published:** 2012-03-30

**Authors:** Carlo Fantoni, Corrado Caudek, Fulvio Domini

**Affiliations:** 1 Center for Neuroscience and Cognitive , Istituto Italiano di Tecnologia, Rovereto, Italy Systems@UniTn; 2 Department of Cognitive, Linguistic & Psychological Sciences, Brown University, Providence, Rhode Island, United States of America; 3 Dipartimento di Psicologia, Università degli Studi di Firenze, Firenze, Italy; University of Muenster, Germany

## Abstract

Humans make systematic errors in the 3D interpretation of the optic flow in both passive and active vision. These systematic distortions can be predicted by a biologically-inspired model which disregards self-motion information resulting from head movements (Caudek, Fantoni, & Domini 2011). Here, we tested two predictions of this model: (1) A plane that is stationary in an earth-fixed reference frame will be perceived as changing its slant if the movement of the observer's head causes a variation of the optic flow; (2) a surface that rotates in an earth-fixed reference frame will be perceived to be stationary, if the surface rotation is appropriately yoked to the head movement so as to generate a variation of the surface slant but not of the optic flow. Both predictions were corroborated by two experiments in which observers judged the perceived slant of a random-dot planar surface during egomotion. We found qualitatively similar biases for monocular and binocular viewing of the simulated surfaces, although, in principle, the simultaneous presence of disparity and motion cues allows for a veridical recovery of surface slant.

## Introduction

The optic flow is an important source of information for the perception of the three-dimensional (3D) structure of the environment [1]–[8]. Our previous research shows that, in passive vision, perceived local surface slant (*i.e.*, the angle between a planar surface and the fronto-parallel plane) is well predicted by a model based on the maximum-likelihood interpretation of the velocity gradient of the optic flow [9]. We also showed that perceived surface slant during ego-motion can be accounted for in a similar manner, with a negligible contribution of extra-retinal signals [10], [11]. Our previous modeling work predicts the perceptual responses from the instantaneous local properties of the optic flow. However, within natural viewing conditions, the velocity gradients vary over time, both when the distal surface is stationary and when it moves within an allocentric frame of reference. It is therefore necessary to understand whether the time variation of the velocity gradients affects the perceptual interpretation of surface slant. In passive vision, we found that perceived slant is indeed influenced by the temporal variation of the velocity gradients [12]–[15]. The purpose of the present study is to determine whether this is also the case in active vision.

To this goal, we measured perceived surface slant in different viewing conditions. In one condition, we simulated a static distal planar surface; depending on the direction of head motion, the velocity gradients of the optic flow either increased or decreased over time. In another condition, we simulated the rotation of a surface in an allocentric frame of reference. In this latter case, the surface rotation was coupled with the amount of head translation, so as to keep the velocity gradients of the optic flow constant over time. The novel result of the present study is that perceived surface slant is biased by the variation of the velocity gradients induced by the motion of the observer's head:

- for a stationary surface in an allocentric frame of reference, observers perceived different surface slants, depending on the magnitude of the velocity gradients, even if the surface was stationary in an earth-fixed reference frame – the velocity gradients were manipulated by simply changing the direction of head translation.
- for a surface that was continuously changing its instantaneous slant within an allocentric frame of reference, observers always reported the same slant magnitude, if the surface rotation was coupled with the head's translation so as to maintain the velocity gradients constant.

### Perceived surface slant in passive vision

Theoretical studies have shown that the second-order temporal properties of the optic flow (*i.e.*, accelerations) are needed to recover veridical surface slant in passive vision [6]–[8], [16]. However, many psychophysical investigations have shown that human observers do not make use of the acceleration components and, therefore, are unable to achieve high levels of veridicality in the perception of surface slant from the optic flow [17], [18]. The empirical research has revealed that perceived surface slant depends almost exclusively on the first-order properties [18]–[25]. Four components can be distinguished in the instantaneous local optic flow: divergence, curl, translation, and deformation [4], [5], [26]. In passive vision, it has been showed that perceived local surface slant is mainly determined by the deformation component (*def*) [17]–[21], [23]–[27,27].

It is important to realize that there is not a one-to-one correspondence between *def* and local surface slant: *def* can vary over time whereas slant can remain unchanged. Nevertheless, empirical evidence indicate that, when *def* varies, so does perceived slant. Therefore, the passively-viewed optic flow is systematically biased by the temporal variation of *def*. This phenomenon has been studied, for example, by [13]. In one experiment, they found that the optic flow induced by a planar surface, which rotates about the vertical axis (*e.g.*, a rigid flag rotating about its post), evokes different perceived slant magnitudes depending on the direction of surface rotation. A surface rotating away from the frontal-parallel plane, which generates an optic flow of pure horizontal contraction, evokes a larger amount of perceived slant than a surface rotating towards the frontal-parallel plane, which generates an optic flow of pure horizontal expansion. In fact, in the first case, *def* continuously increases over time; in the second case, *def* continuously decreases over time. In another set of experiments [14], found that perceived angular velocity of object rotation is strongly affected by the time variation of the median of the distribution of *def* values computed from local patches of the object's surface: If median *def* remains constant over time, so does the perception of angular velocity, regardless of the distal object rotation. Likewise, if median *def* varies over time, so does perceived angular velocity.

In summary, perceived surface slant and perceived object rotation in passive vision are strongly biased by the variation of *def* over time. This does not mean that observers compute a higher-order property, such as the *def* difference in successive moments in time, for example, and then recover surface slant from this property. Instead, the empirical data suggest that observers compute *def* within a very short temporal window [12] and then use *def* to recover surface slant in a heuristic manner [9], [28]. If *def* takes on different values at different moments in time, then perceived surface slant will vary accordingly, regardless of surface slant.

### Perceived surface slant in active vision

More recent theoretical analyses on self-generated (not passively observed) optic flows have shown that, in principle, a veridical reconstruction of the 3D shape and the motion of the visual objects can be achieved if the first-order optic flow is combined with extra-retinal signals resulting from observers self-motion [29]–[42]. An optimal combination of extra-retinal signals and velocity information, however, does not necessarily provide the best model for human active vision [10], [11]. Also in active vision, in fact, dramatic distortions of perceived 3D shape have been found as a consequence of the amount of object rotation and of the head translation velocity, for example [10], [11]. To account for these systematic distortions, we proposed that, also in active vision, perceived surface slant is mainly determined by *def*, whereas extra-retinal information resulting from ego-motion is disregarded [9], [10], [17]–[21], [23]–[27], [27,43]. Our model does not provide a veridical interpretation of the optic flow, because *def* is ambiguous (see Supporting Information S1), but it has been found to be an effective description of perceived surface slant, in both active and passive vision [9]–[11].

**Figure 1:**
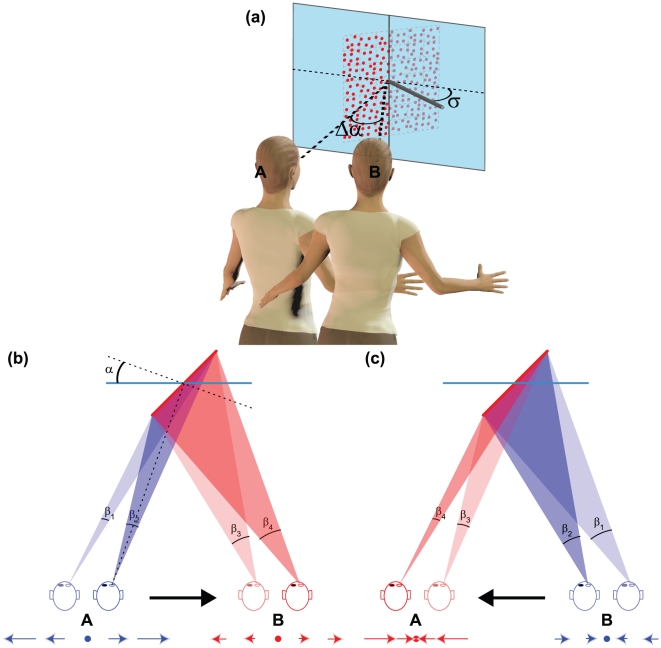
Optic flow variations generated by the relative movement between an observer and a static surface. (a) Schematic representation of the stimuli. A random dot planar surface centered on the image screen (blue transparent plane), was simulated with a slant 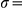 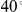 and 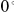 tilt. Tilt is the angle between the x-axis of the image plane and the projection into the image plane of the normal to the surface (grey cylinder). The observer oscillated her head from a position shifted to the left (A) to a position shifted to the right (B) of the planes center. The dashed lines represent the visual directions when the head is in A and B; 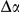 is the variation of the visual direction. The sketches in (b) and (c) shows four successive birds-eye views of the planar surface in (a), with the colors (blue and red) coding for the temporal ordering of the views (initial segment and final segment, respectively). The sketch in (b) illustrates a rightward translation; the sketch in (c) illustrates a leftward translation. The cyan continuous line represents the image screen. The black arrow depicts the direction of head translation. The dashed line in (b) represent the visual direction and its normal through the center of the plane. The *def* component of the optic flow is approximated by the rate of change of the visual angle subtended by the surface. The instantaneous *def* is visualized by the difference between two subsequent visual angles (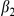
–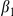, in blue; 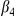
–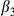, in red). The instantaneous optic flow is depicted below each views segment. The arrows represent the velocity vectors of the optic flow. (b) A rightward head shift induces an optic flow of horizontal expansion with a continuously decreasing *def*. Note indeed that the difference between the two blue subsequent visual angles is larger than the difference between the two red subsequent visual angles. (c) A leftward head shift induces an optic flow of horizontal compression with a continuously increasing *def*. Note indeed that the difference between the two blue subsequent visual angles is smaller than the difference between the two red subsequent visual angles.

In the present study, we develop our previous work by investigating whether the variations of *def* associated with the head movements affects perceived surface slant also within a small temporal window. To address this question, we asked whether a stationary planar surface in an earth-fixed reference frame will be perceived veridically (*i.e.*, with a constant orientation) when the observer moves his head. Observers were instructed to move their head while fixating on a point on a stationary surface oriented with 40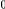 slant and zero tilt (Figure 1a). As indicated in Figure 1b, the lateral translation of the observers head from position A (eccentric to left) to position B (eccentric to the right) produces an optic flow of horizontal expansion. The *def* component of the optic flow corresponds to the rate of this expansion and it can be approximated by the rate of change of the visual angle subtended by the surface (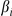). In the specific case represented in Figure 1b, the rate of change is larger in A than in B (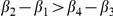). This means that, as the observer moves his head rightward, *def* continuously decreases. The same variation of *def*, but with an opposite temporal ordering, is generated by inverting the direction of the head movement: In this case, *def* is smaller in A than in B (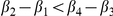). This second case gives rise to an optic flow of horizontal compression with a continuously increasing *def* (see Figure 1c). Note that *def* takes on different magnitudes at the end of the two head translations (from A to B or from B to A), even if the distal surface remains stationary in an allocentric frame of reference. If *def* influences the perceptual interpretation of the optic flow also in active vision, then perceived slant should take on different values depending on whether the observer's head moves rightward or leftward. The current experiments set out to test this hypothesis.

### Experimental design and predictions

**Figure 2:**
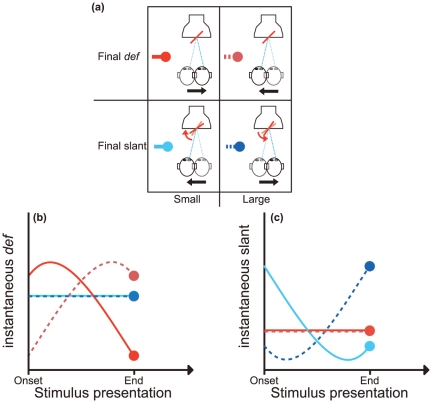
Experimental conditions. (**a**) Bird's eye view of the viewing geometry of the stimulus presentation. Top row: simulation of a planar surface that is stationary in an allocentric frame of reference. Bottom row: simulation of a planar surface that rotates in an allocentric frame of reference. Left column: the translation of the head in the direction of the black arrow and the rotation of the surface in the direction indicated by the curved arrow have the effect of (*i*) decreasing the intensity of *def* while slant remains constant (top: “Small Final *def*”), and (*ii*) decreasing the slant magnitude in an allocentric frame of reference while *def* remains constant (bottom: “Small Final slant”). Right column: the head translation in the direction of the black arrow and the rotation of the surface in the direction indicated by the curved arrow have the effect of (*i*) increasing the intensity of *def* while slant remains constant (top: “Large Final *def*”), and (*ii*) increasing the slant magnitude in an allocentric frame of reference while *def* remains constant (bottom: “Large Final slant”). The red and blue colors code whether, in an allocentric frame of reference, the planar surface was simulated to be stationary or rotating. Light and dark colors code, respectively, the decrease and the increase of the appropriate stimulus property (*def* or slant) during the stimulus presentation. (**b**) The temporal variation of *def* in the four experimental conditions represented in the Panel (a), from the onset to the end of the stimulus presentation. Note that, for an immobile surface in an allocentric frame of reference (Panel a, top row), *def* varies continuously during the translation of the observer (red lines). Note also that the intensity of the instantaneous surface rotations was computed on-line, during the head translation, so as to generate an optic flow with a constant *def* in both the conditions represented in the bottom row of Panel (a). This constant *def* value is represented by the blue lines in Panel (b). (**c**) The temporal variation of the simulated slant magnitudes in an allocentric frame of reference for the four experimental conditions of Panel (a). In the Panels (b) and (c), filled circles indicate the magnitude of *def* (left panel) or slant (right panel) at the end of the stimulus presentation (*i.e.*, the final *def* or final slant, respectively).

In Experiment 1, we simulated a planar surface that was stationary or that rotated with respect to an earth-fixed reference frame. In each trial, the observer translated his/her head either rightward or leftward. Observers judged the slant of the simulated surface immediately after the disappearance of the optic flow generated by their own movement. By combining the simulation of stationary or rotating surfaces with two head-translation directions, we defined the four experimental conditions that are represented in Figure 2a. The top panels illustrate the case in which the simulated surface is stationary. The bottom panels illustrate the case in which the surface rotates.

Figure 2b shows how instantaneous *def* varies in time in the four conditions of Figure 2a. For a stationary surface, a rightward head translation produces the temporal variation of *def* indicated by the light red solid line (see Figure S1 for details). A leftward head translation produces the temporal variation of *def* indicated by the dark red dashed line. As indicated by the light red circle, the value of *def* at the end of a rightward head translation (“final *def*”) is smaller than the value of *def* at the end of a leftward head translation (dark red circle). The two directions of head translation thus define two conditions: a small (rightward) or a large (leftward) final *def*.

In the case of the rotating surface, we updated in real time the angular rotation velocity of the surface as a function of head position, so as to maintain *def* constant during the stimulus presentation. This was achieved by rotating the surface in the counter-clockwise direction around the vertical axis during a rightward head's translation or by rotating the surface in the clockwise direction during a leftward head's translation. The simulated slant of the rotating surfaces, 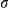, was coupled to the angular speed of the observers head translation, T, and to the visual direction, 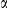, by the equation:
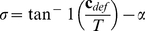
(1)Such a coupling (obtained by inverting the Eq.S1 for 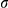 – Supporting Information S1) produced an optic flow with a constant value of *def* corresponding to 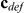. The *def* magnitudes produced by this manipulation are indicated in Figure 2b by the dark and light blue lines. In all four conditions, the average amount of *def* (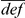) was the same.

Figure 2c shows how the simulated surface slant varies in time with respect to an earth-fixed reference frame in the four conditions of Figure 2a. The light (rightward head translation) and dark (leftward head translation) red lines indicate that the simulated surface slant is kept fixed with respect to an allocentric reference frame in the two conditions illustrated in the top row of Figure 2a. The light and dark blue lines show how slant varies during a leftward (“small final slant”) or rightward (“large final slant”) head translation, respectively. The viewing geometry of these two conditions is illustrated in the bottom row of Figure 2a.

*What should we expect about the perception of surface slant in the four conditions of* *Figure 2a* *?* First, let us consider the case of a stationary surface in an allocentric reference frame (Figure 2a, top row). If perceived slant is determined by the values that *def* takes on at the end of the stimulus presentation (“final *def”*), rather than by 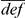, then observers should report a larger surface slant when they perform a leftward rather than a rightward head translation. The “final *def*”, in fact, is larger in the first case than in the second one. This prediction is indicated by the light and dark red circles in Figure 2b.

Now, let us consider a surface that, while rotating with respect to an allocentric reference frame, generates an optic flow with a constant *def* (Figure 2a, bottom row, Figure 2b, light and dark blue lines). Again, if perceived slant depends on *def*, observers should perceive the same slant for rightward or leftward head translations even if the simulates surface slant, in the two cases, is very different at the end of the stimulus presentation (“large final slant” versus “small final slant”) – see the light and dark blue circles in Figure 2c.

These predictions can be contrasted with those deriving from a model that optimally integrates visual and extra-retinal information (*e.g.*, [31]). Such a model always predicts a veridical interpretation of the optic flow, unless there is some systematic error in the measurement of the egomotion or *def* information.

**Figure 3:**
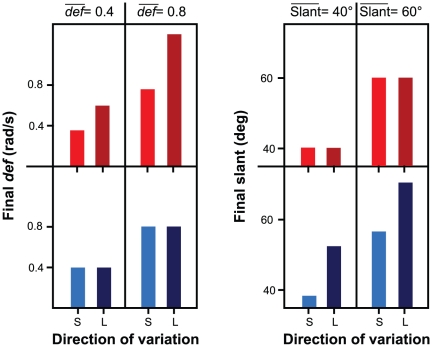
*def* (left panel) and slant (right panel) values at the end of the stimulus presentation. The four cells of each panel reproduce the four experimental conditions represented by the Panel (a) of Figure 2. In the experimental condition represented by each of these cells, the simulated slant took on the values of 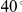 or 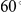 and generated an optic flow having an (average) *def* component equal to 0.4 rad/s or 0.8 rad/s. **Left.** *def* magnitudes at the end of the stimulus presentation as a function of head translation direction (*i.e.*, for the small and large final *def* conditions) for two 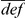 (*i.e.*, 0.4 rad/s or 0.8 rad/s). The color coding is consistent with Figure 2. **Right.** Instantaneous slant in an allocentric frame of reference at the end of the stimulus presentation as a function of head translation direction (*i.e.*, for the small and large final slant conditions) for two 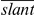 (*i.e.*, 40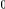 and 60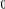). The values shown in the figure have been calculated by considering the stimulus properties of the actual experiments. The left panel illustrates the qualitative predictions of the hypothesis that perceived slant depends on *def*. The right panel illustrates the qualitative predictions of the hypothesis that perceived slant is an unbiased estimate of distal slant.

Figure 2 describes a subset of the conditions that were actually tested in the present study. In two experiments, we generated the optic flows described in Figure 2 for two different simulated slant magnitudes (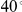 and 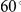). Within our experimental setting, the movement of the observer with respect to a surface with 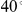 slant generated an average *def* of 0.4 rad/s 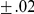; the movement of the observer with respect to a surface with 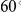 slant generated an average *def* of 0.8 rad/s 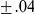. The left panel of Figure 3 shows the “final *def*” in each experimental condition and illustrates the qualitative predictions of the model proposed by [10]. The right panel of Figure 3 shows the amount of simulated surface slant at the end of the stimulus presentation and illustrates the qualitative predictions of a model which optimally integrates the optic flow with the extra-retinal signals resulting from egomotion.

Let us consider the left panel of Figure 3. According to [10], the perceived slant of a stationary surface should be affected both by the direction of the head's translation (“large final *def*” versus “small final *def*”) and by average *def* (top row). Moreover, the effect of the direction of the head's translation should be larger for 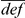 = 0.8 rad/s than for 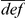 = 0.4 rad/s. Instead, the perceived slant of a rotating surface should be affected by average *def*, but not by the direction of the head's translation (bottom row).

According to a veridical interpretation of the optic flow, which optimally combines retinal information with extra-retinal information resulting from head motion (Figure 3, right panel) the perceived slant of a stationary surface should be affected by the amount of average surface slant (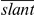), but not by the direction of the head's translation (top row). Instead, the perceived slant of a rotating surface should be affected both by the direction of the head's translation (“large final slant” versus “small final slant”) and by 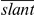 (bottom row).

In Experiment 2, the simulated planar surfaces were defined by motion and by disparity information. According to an inverse geometry approach, when disparity information, version, and vergence signals are added to motion information, the accuracy of the perceptual estimates should increase [44]. Theoretical analyses have shown, in fact, that a correct estimate of surface slant can be recovered from congruent motion and disparity information [45], or from a combination of horizontal disparities, version, and vergence signals [46]–[50] – but see [43], [51], [52]. The model of [10] is agnostic with respect to what should happen to perceived slant when other cues are added to the optic flow. To account for depth cue integration, in our previous research we proposed the Instrinsic Constraint (IC) model [53]–[56]. According to IC, perceived slant, depth, or curvature increase when more cues are added to the stimulus display [57]. This does not mean, however, that the veridicality of the 3D interpretation necessarily increases as well.

To summarize, the hypothesis that perceived slant depends on the instantaneous optic flow, with no contribution of extra-retinal signals in active vision, leads us to expect that (1) *observers will perceive a variable surface slant when they move their head with respect to a stationary surface* (Fig. 2a, top panels), and (2) *observers will perceive a constant surface slant when they move their head with respect to a surface that, while rotating in an allocentric frame of reference, generates a constant def* (Fig. 2a, bottom panels). These predictions can be contrasted with those based on a model which optimally integrates visual and non-visual information.

## Results

### Experiment 1

**Figure 4:**
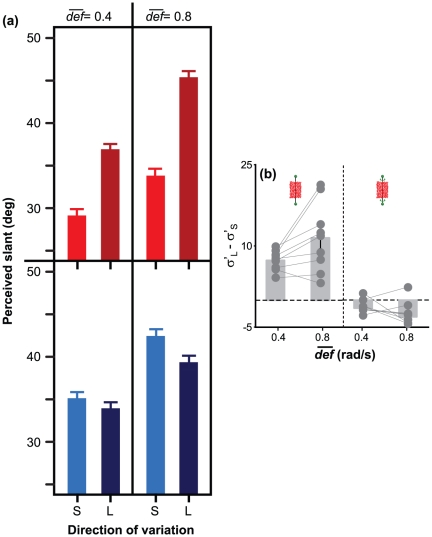
Results of Experiment 1: motion-only information. (a) Average perceived slant in the experimental conditions described in Figure 3. Also the color coding is consistent with that used in Figure 3. (b) Average difference between the responses in the “large” (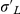) and “small” (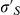) conditions, for both static (left) and rotating (right) planar surfaces, and for 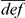 = 0.4 rad/s and 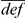 = 0.8 rad/s. Zero indicates no effect of the “large”/“small” manipulation. The dots represent the mean values of the individual observers. In both (a) and (b), vertical bars indicate 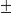
1 S.E. of the mean. Note that these results are consistent with the qualitative predictions of the left panel of Figure 3.

Observers estimated the perceived slant of monocularly-viewed virtual planar surfaces by using a 3D stereo probe. The average amount of perceived surface slant in our experimental conditions is shown in Figure 4.

The results of Experiment 1 are consistent with the predictions of [10]: The interpretation of the self-generated optic flow was determined by *def*, not by simulated slant. Indeed, the slant judgments resemble the pattern of “final *def*” values that are shown in the left panel of Figure 3, not the pattern of “final slant” values that are shown in the right panel of Figure 3. Linear Mixed Effect models with partecipants as random effects, and “final *def*” and the direction of head translation as fixed effects, were used to analyze the slant judgments separately for stationary or rotating planar surfaces in an allocentric frame of reference. We evaluate significance by computing the deviance statistic (minus 2 times the log-likelihood; change in deviance is distributed as chi-square, with degrees of freedom equal to the number of parameters deleted from the model) and with the help of 10,000 samples from the posterior distributions of the coefficients using Markov chain Monte Carlo sampling. From these samples, we obtained the 95% Highest Posterior Density confidence intervals, and the corresponding two-tailed 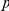-values [58], [59].

For *a surface that was stationary in an allocentric frame of reference*, we found an effects of “final *def*”, 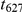 = 8.45, 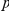 = .001. Overall, the slant estimates were 31% larger in the “large final *def*” condition than in the “small final *def*” condition. We also found an effect of 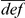, 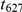 = 11.19, 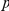 = .001. The slant estimates were 17.5% larger when 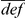 was equal to 0.8 rad/s rather than 0.4 rad/s. The interaction was also significant, 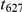 = 5.53, 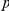 = .001: The difference in the average slant judgments obtained with a leftward or rightward translation was about 17% larger when 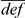 = 0.8 rad/s rather than 0.4 rad/s. These results indicate that observers perceive an horizontally-tilted surface as having different slants if they perform a rightward or a leftward head translation. The magnitudes of perceived slant judgments are consistent with the magnitudes of “final *def*.”

For *a surface that rotated in an allocentric frame of reference*, we found an effect of 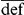, 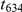 = 9.50, 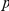 = .001 (Fig. 4a, bottom panels), but not of “final slant” 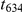 = 1.64, 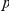 = .1. The interaction term was not significant, 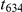 = 1.95, 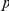 = .06. These results indicate that a surface, which rotates within an allocentric frame of reference, can be perceived as having the same slant in the different moments of the rotation if *def* remains constant (Fig. 4a, bottom panels, Fig. 3, blue bars).

In summary, the results of Experiment 1 follow the same qualitative trend that has been found in passive vision. They indicate that slant judgements strongly depend on *def* and provide no evidence that extra-retinal signals from head movement contributes to the perceptual response beyond what *def* can explain.

### Experiment 2

**Figure 5:**
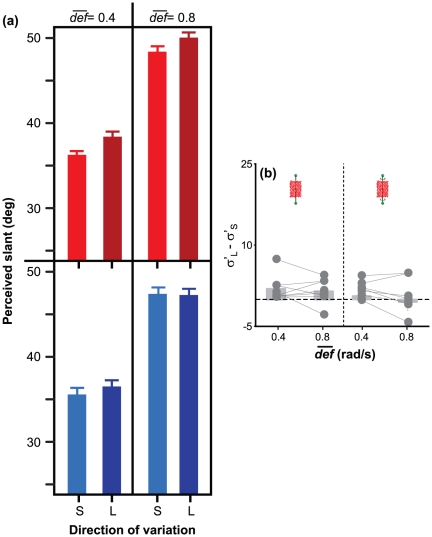
Results of Experiment 2: motion+disparity information. (a) Average perceived slant in the experimental conditions described in Figure 3. (b) Average difference between the responses in the “large” (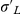) and “small” (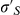) conditions, for both static (left) and rotating (right) planar surfaces, and for 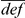 = 0.4 rad/s and 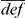 = 0.8 rad/s. The dots represent the mean values of the individual observers. In both (a) and (b), vertical bars indicate 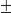
1 S.E. of the mean.

In Experiment 2, the stimuli and procedure were the same as in Experiment 1, but viewing was binocular. The optical information comprised both optic flow and binocular disparity; extra-optical information included vestibular and proprioceptive information about head movements, version and vergence signals. Despite the richer stimulus information, the results of Experiment 2 are similar to those of Experiment 1 (see Figure 5).

For *a surface that was stationary in an allocentric frame of reference*, perceived slant was larger when “final *def*” was larger (Figure 5a, top panels), 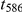 = 4.52, 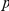 = .001. A stationary surface was thus perceived as having different slants, depending on whether the observer translated his/her head leftward or rightward. Perceived slant was also affected by 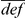, 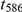 = 17.33, 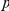 = .001: the larger 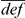 the larger the amount of perceived slant.

For *a surface that rotated in an allocentric frame of reference* so as to generate a constant *def*, perceived slant was not affected by “final slant”, 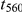 = 1.1, 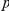 = .27 (Figure 5a, bottom panels). Perceived slant was affected by 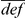 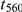 = 18.42, 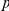 = .001. In conclusion, also in Experiment 2 the judgments of surface slant were biased by 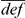 and by “final *def*”, even though the simulated surfaces were rendered with consistent stereo and motion cues.

### Comparison of Experiments 1 and 2

A measure of bias induced by *def* on perceived slant can be provided by the difference between the slant judgments in the “large final *def*” condition (leftward head translation) and in the “small final *def*” condition (rightward head translation). The size of this bias decreases when binocular disparity is added to the optic flow: Note that the bars shown in the left panel of Figure 5b are smaller than those in the left panel of Figure 4b. In Experiment 1, the average bias was equal to 9.7 (SD = 7.98). In Experiment 2, this bias decreased by 7.8, 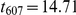, 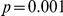, but it was still significantly larger than zero: On average, it was equal to 1.8, 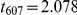, 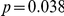. By looking at the Figures 4b and 5b, it is also clear that, by adding binocular disparity to the optic flow, the amount of perceived slant increased. On average, perceived surface slant was 5.6 larger in Experiment 2 than in Experiment 1 for static surfaces, 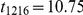, 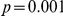, and 2.5 larger in Experiment 2 than in Experiment 1 for rotating surfaces, 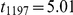, 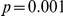.

## Discussion

In the present investigation, we asked whether and to what degree the variation of *def* over time, which is caused by the movement of the observer's head, biases the perception of surface slant. For stationary surfaces in an allocentric frame of reference, the model proposed by [31] predicts no effect of *def* on perceived surface slant. The model proposed by [10], instead, predicts that perceived surface slant will take on different values, if *def* takes on different values, regardless of the distal surface slant. Our results support this second hypothesis. We found that (1) observers perceived different surface slants, depending on the magnitude of *def* at the end of the head's translation, even if the distal surface remained immobile in an allocentric frame of reference, and (2) observers perceived a constant amount of surface slant, when *def* was kept constant, even if the distal surface changed continuously its slant over time in an allocentric frame of reference.

Many researchers have suggested that the self-generated optic flow is advantageous for robust and veridical 3D perception over the passively-viewed optic flow. The ambiguities in the perception of tilt that are present in passive vision, for example, can be resolved when the optic flow is generated by the movement of the observer [32], [35], [36]. These results have been taken to mean that, for disambiguating the optic flow, the human visual system takes into account the extra-retinal ego-motion signals, in an analysis that is consistent with an inverse-optics approach ([31]–[36], [41], [60]–[62], but see [9], [22], [63], [64]). The results of the present work, together with our previous studies, do not support this view [10], [11]: Also when extra-retinal information resulting from ego-motion is available, the perception of surface slant is strongly biased by *def*.

To reconcile our results with those in the literature, it is necessary to distinguish between two distinct problems: the recovery of surface tilt from the optic flow and the recovery of surface slant. The recovery of surface tilt requires the computation of affine relationships between object points [43], [65], [66], whereas the recovery of surface slant requires the knowledge of Euclidean 3D properties [37]. In general, if the extra-retinal information resulting from ego-motion is not available, surface slant remains underdetermined, but surface tilt can be recovered up to a 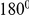 reflection. To determine the orientation of a surface is necessary to specify both tilt and slant, but most investigations supporting the role of extra-retinal information in the perceptual interpretation of the optic flow have focused on the perception of tilt. The contribution of the present study is to show that the variation of *def* over time, which is caused by the movement of the observer's head, can systematically bias the perceptual recovery of surface slant, even for surfaces that are stationary in an allocentric frame of reference (Experiment 1). We also found that the biases induced by the variation of *def* over time persist, in a reduced form, also when binocular disparity is added to the stimulus displays (Experiment 2).

The systematic distortions of perceived surface slant that we describe in the present study resemble those that we had previously found in passive vision [12]–[15]. In the present study, we did not replayed to the passive observer the optic flows that had been generated by the motion of the observer's head (*e.g.*, [11]), so we cannot determine whether the presence of extra-retinal information reduces the magnitude of the *def*-induced biases. However, we found that these biases were strongly reduced when binocular disparity was added to the stimulus displays. If we consider the case of a stationary surface in an allocentric frame of reference, the biases in perceived surface slant can be quantified by the difference between the average slant judgments obtained in a rightward and a leftward head translation. As indicated in Figures 5b and 4b, the *def*-induced bias is 77% smaller when binocular disparity is added to the optic flow. According to [45], the simultaneous presence of the disparity and motion cues provides an additional constraint that can be used for a veridical reconstruction of 3D slant. Moreover, binocular viewing provides non-visual information (like vergence and version) that, in principle, can be used to improve the interpretation of the optic flow [46]–[50]. While it is reasonable to expect that the accuracy of slant estimation increases when adding disparity information, it is surprising that the richer stimulus information of Experiment 2 does not eliminate the biases in perceived surface slant completely.

Another consideration concerns the fact that the amount of perceived slant increased by 14% when binocular disparity was added to the actively-generated optic flow. This result can be interpreted in two ways. (1) It is consistent with the IC model, which hypothesizes that stimuli with a larger number of depth cues support a larger amount of perceived slant. According to IC, perceived surface slant is estimated in an heuristical manner as a monotonic function of the combination of the image signals that maximizes the accuracy of the recovered affine structure [53]–[56]. Therefore, the magnitude of perceived slant is expected to increase if the number of depth cues increases [57]. (2) It is consistent with the hypothesis that the perceptual solution improves (*i.e.*, becomes more veridical) as more information is added to the stimulus displays [44], [67], [68]: The larger amount of perceived slant in Experiment 2 provides a better approximation of the veridical solution than the amount of slant perceived in Experiment 1. It remains a problem of future research to determine whether the increase of the perceived slant magnitudes found in Experiment 2 is better explained by a probabilistic model (such as the IC model), which does not necessarily converge toward the veridical solution, or by an “inverse optics” model, which optimally combines retinal and extra-retinal information.

### Conclusions

The results of the present study suggest that the perceptual recovery of surface slant from the optic flow is affected by systematic biases, in both active and passive vision. These systematic biases are well explained by the model proposed by [9]–[11]. Interestingly, these biases persist, even though in a reduced form, also under realistic conditions in which the observer moves at a normal speed past a surface while binocularly fixating one of its points, when sufficient information is available for an unbiased estimate of surface slant [31].

## Materials and Methods

### Participants

Seventeen undergraduate students of the University of Trento participated in the experiments: nine in Experiment 1 and eight in Experiment 2. All of them were naïve to the purpose of the experiment. All subjects had normal or corrected-to-normal vision. They were paid for their participation. All experiments were undertaken with the understanding and written consent of each subject, with the approval of the *Comitato Etico per la Sperimentazione con lEssere Umano* of the University of Trento, and in compliance with national legislation and the Code of Ethical Principles for Medical Research Involving Human Subjects of the World Medical Association (Declaration of Helsinki).

### Apparatus

The participants head motions were recovered in real-time by an Optotrak 3020 Certus system. A Dell Precision T3400 525W (using an Intel Core 2 Extreme 5252W, QX9650, 3.00 GHz, 1333 MHz FSB,12 MB L2 Cache) controlled the stimulus display and sampled the tracker (using a standard PCI card). Three sensors on the back of the observers head were used to calculate the 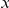, 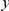, 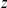 coordinates of the observers viewpoint in order to update in real time the geometrical projection of a Random-Dot planar Surface (RDS). The positions of RDS forming our stimuli were updated on a ViewSonic 9613, 19W CRT monitor. The monitor was set at a resolution of 1024
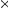
768 pixels (0.24-mm diagonal dot pitch) and was driven by an nVidia Quadro FX 4600 with 768 Mb. The refresh rate of the monitor was 120 Hz.

**Figure 6:**
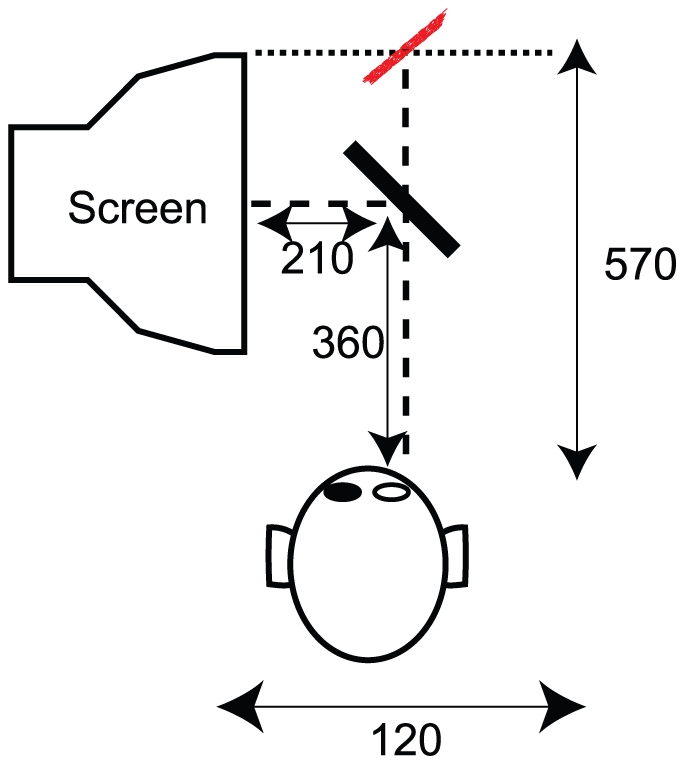
Viewing apparatus and setting. A bird-eye view of the viewing apparatus and of the experimental setting, including the mirror, the CRT screen, the observer. The simulated slanted plane is represented by the red dots. Dashed lines show the light path, from the CRT to the lumen of the eye, for a standard observer at rest. Distance units are expressed in mm.

The stimuli were viewed through a high-quality front-silvered mirror (400
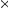
300 mm) placed at eye-height in front of the observers central viewing position and slanted 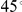 away from the monitor and the observers inter-ocular axis. As shown in Figure 6, the screen distance from the center of the mirror was 210 mm, while, on average, the distance from the pupil to the center of the mirror was 360 mm. This arrangement produced an average viewing distance (i.e., distance between the pupil and the center of the display as reflected by the mirror) of 570 mm.

A custom Visual C++ program supported by OpenGL libraries and Optotrak API routines was used for stimulus presentation and response recording. The same program controlled the slant of the RDS, depending on observers translation velocity, according to Eq. 1.

Displays were viewed through liquid-crystal-diode (LCD) shutter glasses synchronized to the monitor (FE-1 Goggles, Cambridge Research System). Shutter glasses were used to control monocular and binocular presentation of our displays. As a consequence of the use of the shutter glasses, the actual refresh rate was 60 Hz.

### Displays

The displays were random arrangements of (1
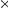
1 mm) 300 antialiased red dots. The motion of these dots simulated the optical velocity field generated by a planar surface slanted around the vertical with 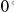 tilt and centered on the image screen. Each trial included a sequential presentation of two stimuli: a test stimulus, shown during observers head translation, and a probe stimulus. The probe was always viewed binocularly from a static central position with the cyclopean line of sight centered and aligned to the screen center.

Non-motion cues, such as texture or outline foreshortening, were removed from the test display, but not from the probe display. To do so we determined the dots distribution using a back-projection technique [69]. Dots were randomly distributed in the projected image, not on the simulated surface. This was achieved by imposing 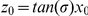 , with 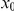 and 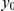 randomly selected in the range between 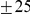 mm from the screen center. The stimulus onset asynchrony between the test and the probe displays was sufficiently large to avoid any backward masking effect (500 msec). The probe display was a stationary random-dot planar surface defined by binocular disparity information, texture gradients, and outline foreshortening. An appropriate key-press allowed participants to adjust the simulated slant of the probe surface whereas the tilt was kept constant at 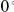.

For each stimulus frame, the dots of the test and the probe surfaces were projected onto the screen by using a generalized perspective pinhole model with the observer's eyes position (measured with almost no latency) used as center of projections. In both the probe and test displays, the motion of the dots that was induced by the relative motion between the simulated surface and the observer generated an approximately linear optic flow with horizontal velocity vectors (see Figure 1).

The test stimulus was visible while the observer moved his/her head. The onset of the test stimulus occurred when the right eye crossed a position 60 mm eccentric to the left (A) or to the right (B) of the center of the screen (i.e., when the right eye was on the plane orthogonal to the screen vertical midline), after the observer reversed his direction of motion, which occurred somewhat after the right eye was in A or B. The test stimulus was displayed either after the eye crossed the position A, during a rightward head translation (horizontal expansion of the optic flow), or vice-versa, after the eye crossed the position B during a leftward head translation (horizontal compression of the optic flow). At the average velocity of 240
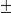
80 mm/s, the test stimulus was visible on the screen for about 0.5 sec. The test stimulus was deleted when the right eye crossed the eccentric position (A) or (B) opposite to that of stimulus onset.

In the case of the stationary surfaces, we simulated a static planar surface centered on the image screen with a slant of either 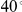 or 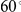. The viewing distance was 570 mm. The lateral head shift was equal to about 120 mm and the consequent variation of the visual direction produced an optical angle of about 13. When the surface was simulated to be static in an allocentric reference frame, during the head translation, the relative slant of the surface with respect to the observers optical axis varied between: 33.5 (position A) and 46.5 (position B), for the 40 slanted surface, and between 53.5 and 66.5, for the 60 slanted surface. As a consequence, the width of the projected image varied from 45 mm to 55 mm, for the 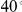 slanted surface, and from 39 mm to 61 mm, for the 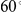 slanted surface. The average *def* for the 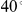 and the 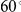 slanted surfaces was equal to 0.4
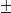
0.02 rad/s and 0.8
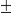
0.04 rad/s, respectively (see Figure S1, panel c, for the average *def* values produced by a representative subject performing a rightward head translation).

In the case of the rotating distal surfaces, we simulated the projection of a planar surface centered on the image screen. Such planar surfaces was simulated as rotating during the head translation, so as to generate a constant value of *def* of either 0.4 rad/s or 0.8 rad/s. The real-time update of the simulated surface slant, as a function of the observersposition and velocity, produced a variation of the simulated slant of about 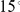. The average slant of the surfaces generating a *def* of 0.4 rad/s or 0.8 rad/s was equal to 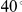 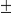 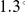 or 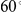 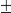 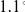, respectively. The variation of the simulated slant varied the width of the projected image from 45 mm (when in A) to 59 mm (when in B) in the case of *def* equal to 0.4 rad/s, and from 40 mm to 72 mm, in the case of *def* equal to 0.8 rad/s.

In Experiment 2, the test displays were viewed binocularly and thus included a disparity slant cue that was the difference between left-and right-eye projections of corresponding surface points, calculated separately for each observers inter-ocular distance. In this condition, the stimulus was defined by the same random dot textures described above.

### Procedure

Participants were tested individually in complete darkness, so that only the stimuli were visible during the experiment. In particular, the monitor's frame and the mirror were not visible. To allow for natural head movements, during the experiment the head was not restrained. Prior to the experiment, each participant was trained to perform back-and-forth lateral head translations at the required velocity of 240
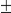
80 mm/s when crossing the center of the screen. Participants were also instructed to minimize head rotations as well as movements in the vertical and depth direction.

**Figure 7:**
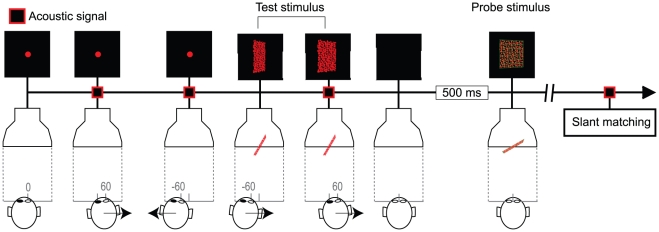
Schematic representation of the temporal sequence of stimulus events. The figure illustrates the situation corresponding to the simulation of a static surface which induces a decreasing *def* when combined with a rightward head translation.

At the beginning of each trial, a fixation mark was shown in the center of the screen and participants were required to align their right eye with the fixation mark (Figure 7). If the head position was not within 5 cm of an “ideal” starting position located at 570 mm from the monitor screen, then the fixation mark was painted in green, thus signaling a misplacement of the head. When the participant's head was correctly positioned, the fixation mark turned red and the participant moved her head rightward. The direction of head motion reversed when a beep signaled a head shift of 60 mm relative to the center of the screen. The acoustic signals also provided a feedback about the speed of the head translation: a high-pitch sound signalled a speed that was too fast, a low-pitch sound signalled a speed that was too slow. During the first oscillation cycles, the stimulus display was not shown. The test surface was displayed after three and half head oscillation cycles in the horizontal plane at the required velocity and at the required head orientation (i.e., yaw, pitch, and roll were controlled in real time and were required to be within the 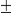 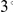 range). The test stimulus remained visible for an entire oscillation cycle (about 0.5 s). After the test display disappeared, the probe stimulus was shown. The time separating the test stimulus and the probe stimulus was 0.5 s. The participants' task was to adjust the simulated slant of the probe stimulus, so as to match the perceived slants of the test and of the probe surfaces. During the execution of this task, the participants did not move their head.

For both Experiments 1 and 2, the experimental session lasted for about 90 min and comprised two blocks of 80 trials each. In each block the simulated surfaces were either stationary or rotating. The ordering of the two blocks was counter-balanced across participants. Each block consisted of 20 random sequences of the four experimental conditions: 2 average magnitudes of *def* (0.4 rad/s and 0.8 rad/s, corresponding to an average surface slant of 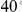 and 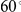 respectively) 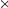
2 head translation directions (rightward and leftward). Before each block of trials, participants received a brief training to familiarize them with the task and the stimuli.

## Supporting Information

Supporting Information S1 Instantaneous *def* and its relationship with lateral head translation.
(PDF)

Figure S1
**Co-variation between** ***def*** **and lateral head position.** In our experiments, observers performed a sinusoidal lateral head translation while fixating a 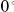 tilted planar surface. Panel (a) shows the variation of the head angular velocity during the oscillatory head translation. Panel (b) shows the variation of the relative slant of the surface. The relative slant 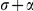 is the angle between the surface and the orthogonal to the viewing direction. Panel (c) shows the variation of *def* during the oscillatory head translation. The curves shown in the figure have been computed by assuming the actual viewing parameters used in the experiments (viewing distance of 570 mm, head position range between 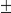
120 mm, average translation velocity of 240 mm/s). A top view of the head positions is shown below the 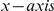. The cyan dashed lines indicate the viewing direction and its orthogonal dimension. The average (normalized) values of a representative subject are indicated by the red insets from the onset to the offset of the stimulus.(EPS)
