# Supplementary material for: Perceived Surface Slant Is Systematically Biased in the Actively-Generated Optic Flow
Source: PLoS One. 2012 Mar 30;7(3):e33911. doi: 10.1371/journal.pone.0033911 (PMC3316515; doi:10.1371/journal.pone.0033911)
Supplement: Supporting Information S1 — Instantaneous def and its relationship with lateral head translation. (PDF) [file pone.0033911.s001.pdf]

Supporting Information

When the observer's moves relative to a static surface slanted around the vertical (Figure 1), the visual direction, α, change in time. This variation of α is defined by the angular velocity T. Fantoni et al [1] have shown that the relationship between T, α, and the slant of the surface (σ) is well approximated by the following equation:

$$def = T \tan(\sigma + \alpha) \tag{S1}$$

Eq.S1, clarifies the ambiguity of *def* [1–3]. If extra-retinal signals are ignored, T and α are left unspecified and *def* remains ambiguous, not only in each single moment in time but also across an extended time window. Different combinations of T, α, and σ, in fact, can produce to the same *def* value.

The relationship between lateral head translation and *def* is clarifyed by Figure S1. The head velocity profile (i.e., T) induced by a sinusoidal back-forth lateral head translation similar to the one used in our experiments is shown in the panel (a). The same lateral translation of the head causes a variation of the relative slant between the surface and the line of sight (i.e., $(\sigma + \alpha_i)$), which is shown in the panel (b). Relative slant increases or decreases with respect to the actual slant of the surface depending on whether the head is translating to the right or to the left of the surface center. The instantaneous *def* profile resulting from the combination of the head translation velocities, shown in (a), and the relative slant values, shown in (b), is depicted in panel (c).

References

1. Fantoni C, Caudek C, Domini F (2010) Systematic distortions of perceived planar surface motion in active vision. Journal of Vision 10: 1–20.

2. Freeman TCA, Fowler TA (2000) Unequal retinal and extra-retinal motion signals produce different perceived slants of moving surfaces. Vision Research 40: 1857 – 1868.

3. Koenderink JJ, van Doorn AJ (1978) How an ambulant observer can construct a model of the environment from the geometrical structure of the visual inflow. Kybernetik : 224–247.

Figure Legends

Figure S1. Co-variation between *def* and lateral head position. In our experiments, observers performed a sinusoidal lateral head translation while fixating a 0° tilted planar surface. Panel (a) shows the variation of the head angular velocity during the oscillatory head translation. Panel (b) shows the variation of the relative slant of the surface. The relative slant $\sigma + \alpha$ is the angle between the surface and the orthogonal to the viewing direction. Panel (c) shows the variation of def during the oscillatory head translation. The curves shown in the figure have been computed by assuming the actual viewing parameters used in the experiments (viewing distance of 570 mm, head position range between ± 120 mm, average translation velocity of 240 mm/s). A top view of the head positions is shown below the $x - axis$. The cyan dashed lines indicate the viewing direction and its orthogonal dimension. The average (normalized) values of a representative subject are indicated by the red insets from the onset to the offset of the stimulus.
